# Supplementary figures and images for: Commensal bacteria augment Staphylococcus aureus infection by inactivation of phagocyte-derived reactive oxygen species
Source: PLoS Pathog. 2021 Sep 16;17(9):e1009880. doi: 10.1371/journal.ppat.1009880 (PMC8478205; doi:10.1371/journal.ppat.1009880)

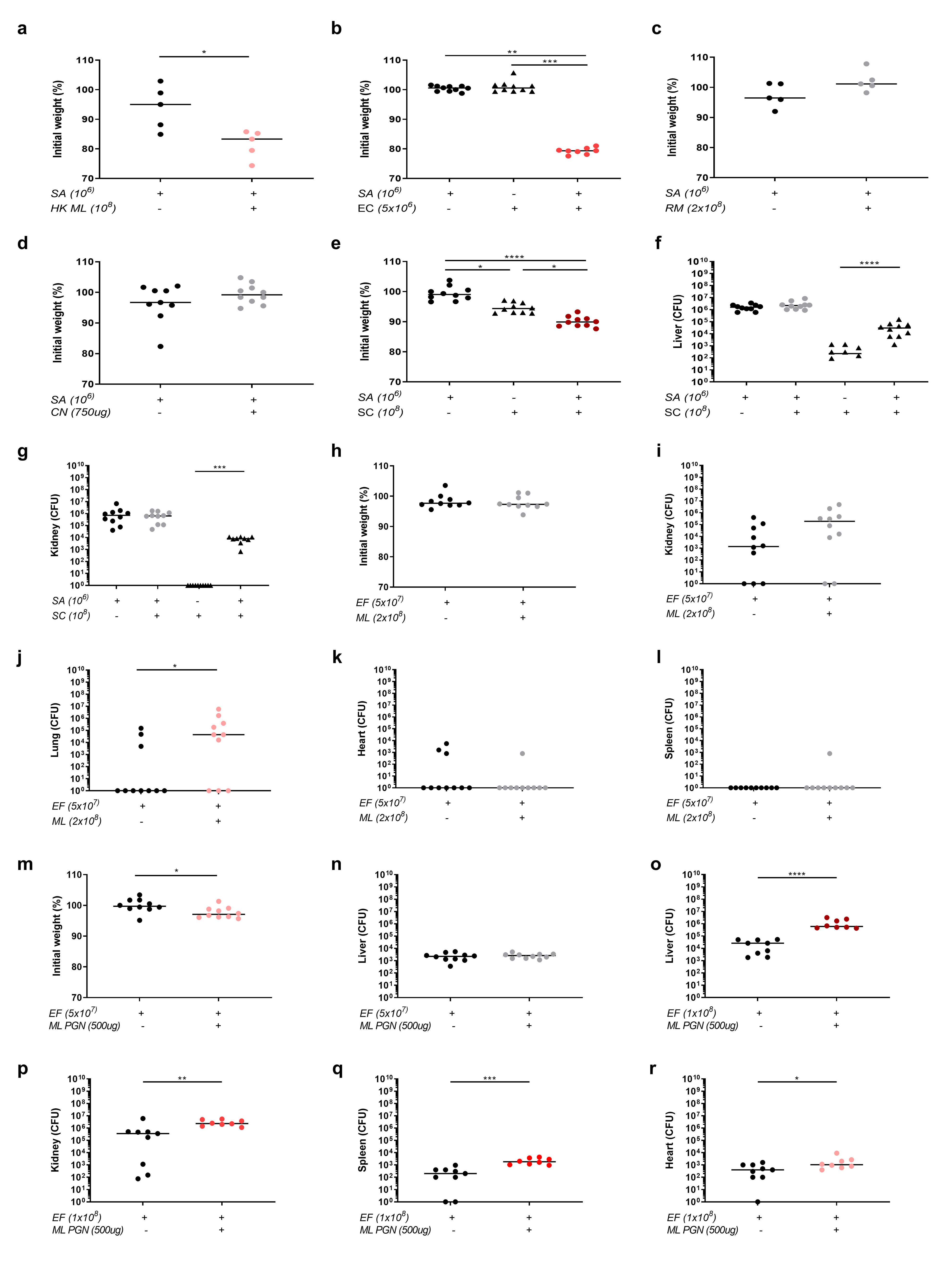

Supplement: S1 Fig — A Co-injection of low dose S. aureus (SA 1x10^6^ CFU) with heat-killed M. luteus (HK ML equivalent of 1x10^8^ CFU) into mice: weight loss (n = 5 per group), *p<0.05 B Co-injection of low dose S. aureus (SA 1x10^6^ CFU) with E. coli (EC 5x10^6^ CFU) into mice: weight loss (n = 8–10 per group, S. aureus, circles; E. coli triangles), **p<0.003; ***p<0.0005 C Co-injection of low dose S. aureus (SA 1x10^6^ CFU) with R. mucosa (RM 2x10^8^ CFU) into mice: weight loss (n = 5 per group) D Co-injection of low dose S. aureus (SA 1x10^6^ CFU) with heat-killed C. neoformans (CN 750 μg) into mice: weight loss (n = 9–10 per group) E-G Co-injection of low dose S. aureus (SA 1x10^6^ CFU) with S. cerevisiae (SC 1x10^8^ CFU) into mice, (n = 10 per group, S. aureus, circles; S. cerevisiae triangles): E weight loss, *p<0.05; ****p<0.0001, F liver CFU, ****p<0.0001, G kidney CFU, ***p<0.0005 H-L Co-injection of low dose E. faecalis (EF 5x10^7^ CFU) with M. luteus (ML 2x10^8^ CFU) into mice: H weight loss (n = 10 per group), I kidney CFU, J lung CFU, *p≤0.05, K heart CFU, L spleen M-N Co-injection of low dose E. faecalis (EF 5x10^7^ CFU) with M. luteus PGN (ML PGN 500 μg) into mice (n = 10 per group): M weight loss, *p<0.05, N liver CFU, O-R Co-injection of high dose E. faecalis (EF 1x10^8^ CFU) with M. luteus PGN (ML PGN 500 μg) into mice, CFUs taken at 48 hpi (n = 8–9 per group): O liver CFU, ****p<0.0001, P kidney CFU, **p<0.0.003, Q spleen CFU, ***p<0.0.0005, R heart CFU, *p<0.05. Colours used indicate the level of significance as indicated in Fig 1E. For all panels, the median value is shown. For panels B and E, a Kruskal-Wallis test with Dunn’s post-test was used, for all remaining panels a two-tailed Mann-Whitney test was used. CFUs were enumerated at 3 days post-infection, unless otherwise stated. (TIF) [file ppat.1009880.s001.tif]

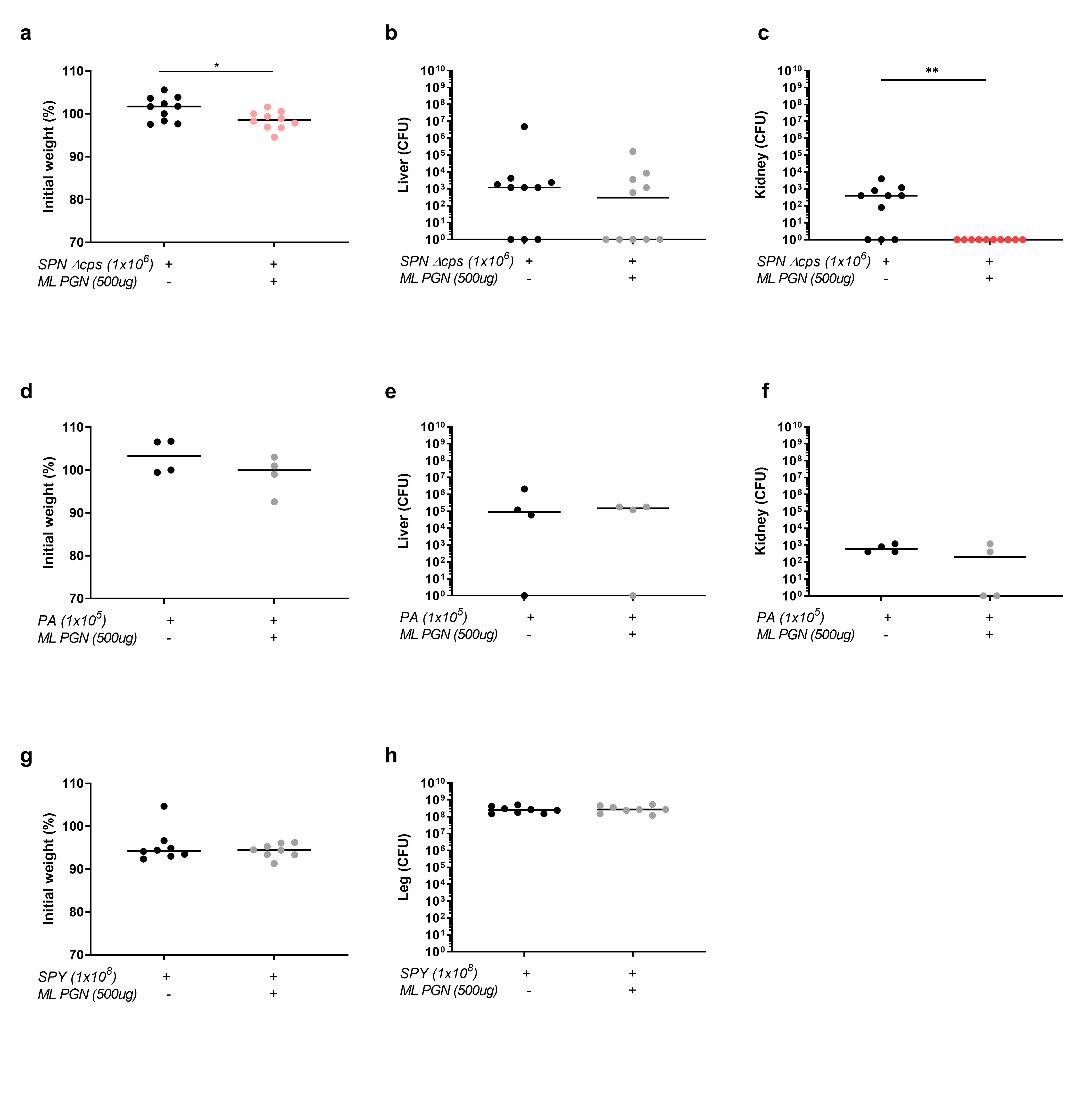

Supplement: S2 Fig — A-C Co-injection of low dose S. pneumoniae (SPN 1x10^6^ CFU) with M. luteus PGN (ML PGN 500 μg) into mice (n = 10 per group), CFUs enumerated at 3 days post-infection: A weight loss, *p<0.05, B liver CFU, C kidney CFU, **p<0.003 D-F Co-injection of low dose P. aeruginosa (PA 1x10^5^ CFU) with M. luteus PGN (ML PGN 500 μg) into mice (n = 10 per group), CFUs enumerated at 3 days post-infection: D weight loss, E liver CFU, F kidney CFU G-H Intramuscular co-injection of S. pyogenes (SPY 1x10^8^ CFU) with M. luteus PGN (ML PGN 500 μg) into mice (n = 8 per group): G leg hindlimb CFU at 24 hpi, H weight loss at 24 hpi. Colours used indicate the level of significance as indicated in Fig 1E. For all panels, a two-tailed Mann-Whitney test was used and the median value is shown. (TIF) [file ppat.1009880.s002.tif]

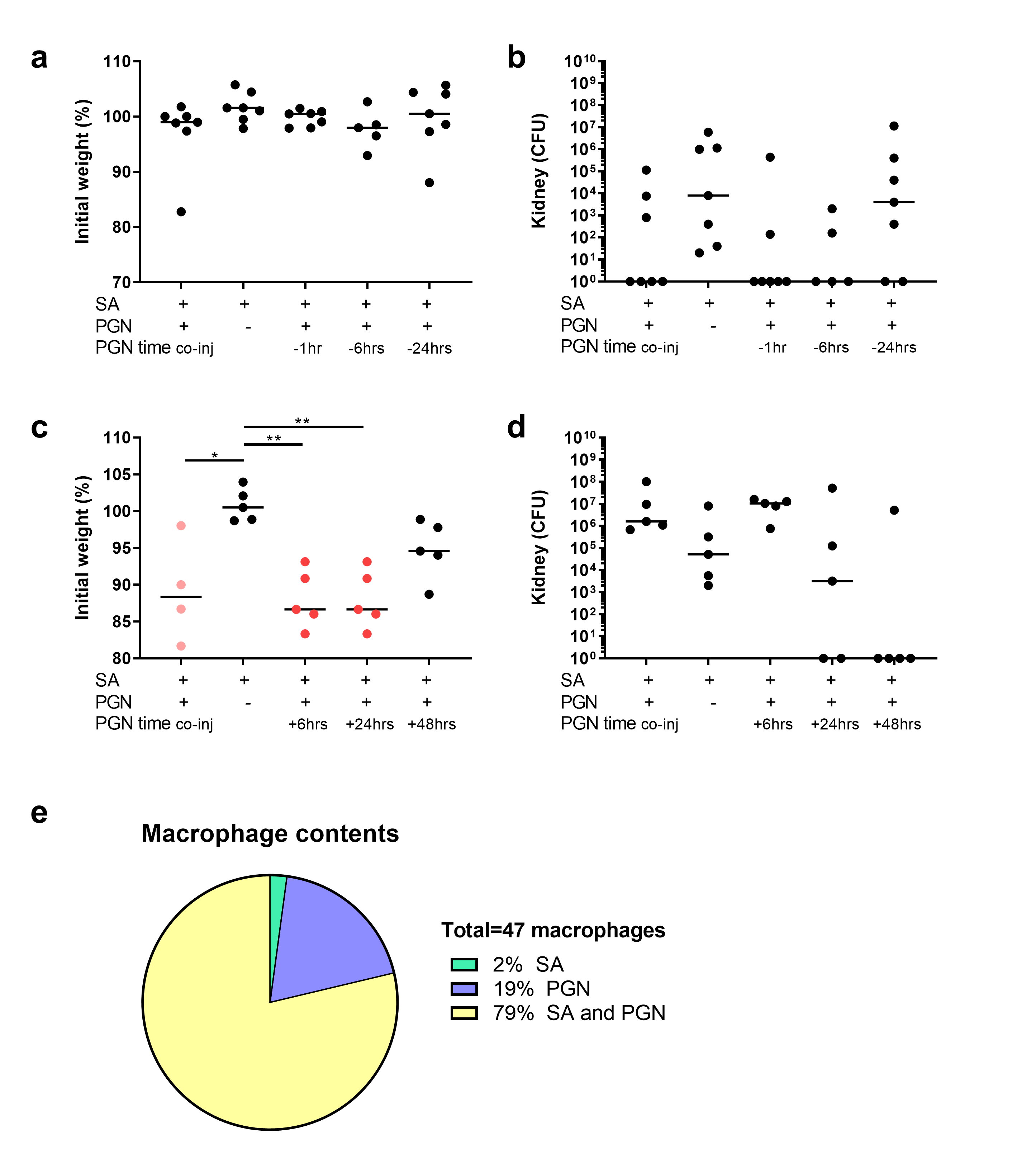

Supplement: S3 Fig — A-B Mice were intravenously injected with 500 μg M. luteus PGN 24 hours, 6 hours or 1 hour before infection with 1x10^6^ S. aureus, at the same time as S. aureus, or with S. aureus alone (n = 7 per group, median value shown, Kruskal-Wallis tests with Dunn’s post-test) A weight loss B kidney CFU C-D Mice were intravenously injected with 500 μg M. luteus PGN 48 hours, 24 hours or 6 hours after infection with 1x10^6^ S. aureus, at the same time as S. aureus, or with S. aureus alone (n = 5 per group, median value shown, Kruskal-Wallis tests with Dunn’s post-test) C weight loss, *p<0.05; **p<0.008 D kidney CFU E quantitation of zebrafish macrophages from Fig 2I, showing the percentage containing S. aureus only (green), PGN only (blue) or S. aureus co-localising with PGN (yellow) (n = 47 macrophages from 21 larvae). (TIF) [file ppat.1009880.s003.tif]

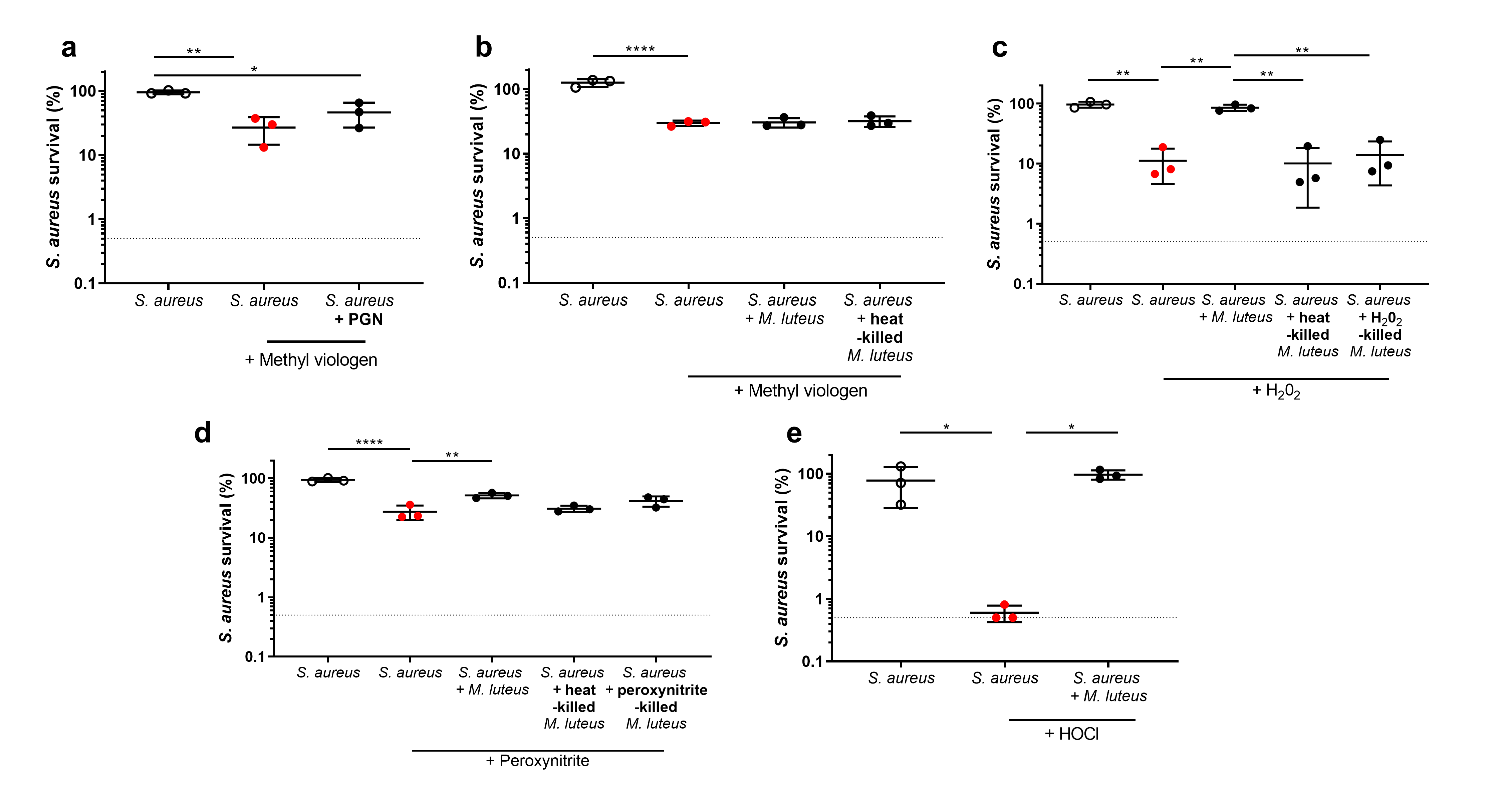

Supplement: S4 Fig — A Following methyl viologen exposure, percentage survival of S. aureus alone (5x10^4^ CFU/mL), or with M. luteus PGN (1.25 mg) (n = 3, error bars show mean +/- SD, one-way ANOVA test with Tukey’s post hoc test), *p<0.05; **p<0.01 B-D Following ROS exposure, percentage survival of S. aureus alone (5x10^4^ CFU/mL), or with live M. luteus (5x10^6^ CFU/mL), heat-killed M. luteus (equivalent of 5x10^6^ CFU/mL) or ROS killed M. luteus (equivalent of 5x10^6^ CFU/mL), (n = 3, error bars show mean +/- SD, one-way ANOVA tests with Tukey’s post hoc test) B methyl viologen, ****p<0.0001 C hydrogen peroxide, **p<0.007 D peroxynitrite, **p<0.003; ****p<0.0001 E Following sodium hypochlorite exposure, percentage survival of S. aureus alone (5x10^4^ CFU/mL), or with live M. luteus (5x10^6^ CFU/mL), (n = 3, error bars show mean +/- SD, one-way ANOVA test with Tukey’s post hoc test), *p<0.05. (TIF) [file ppat.1009880.s004.tif]

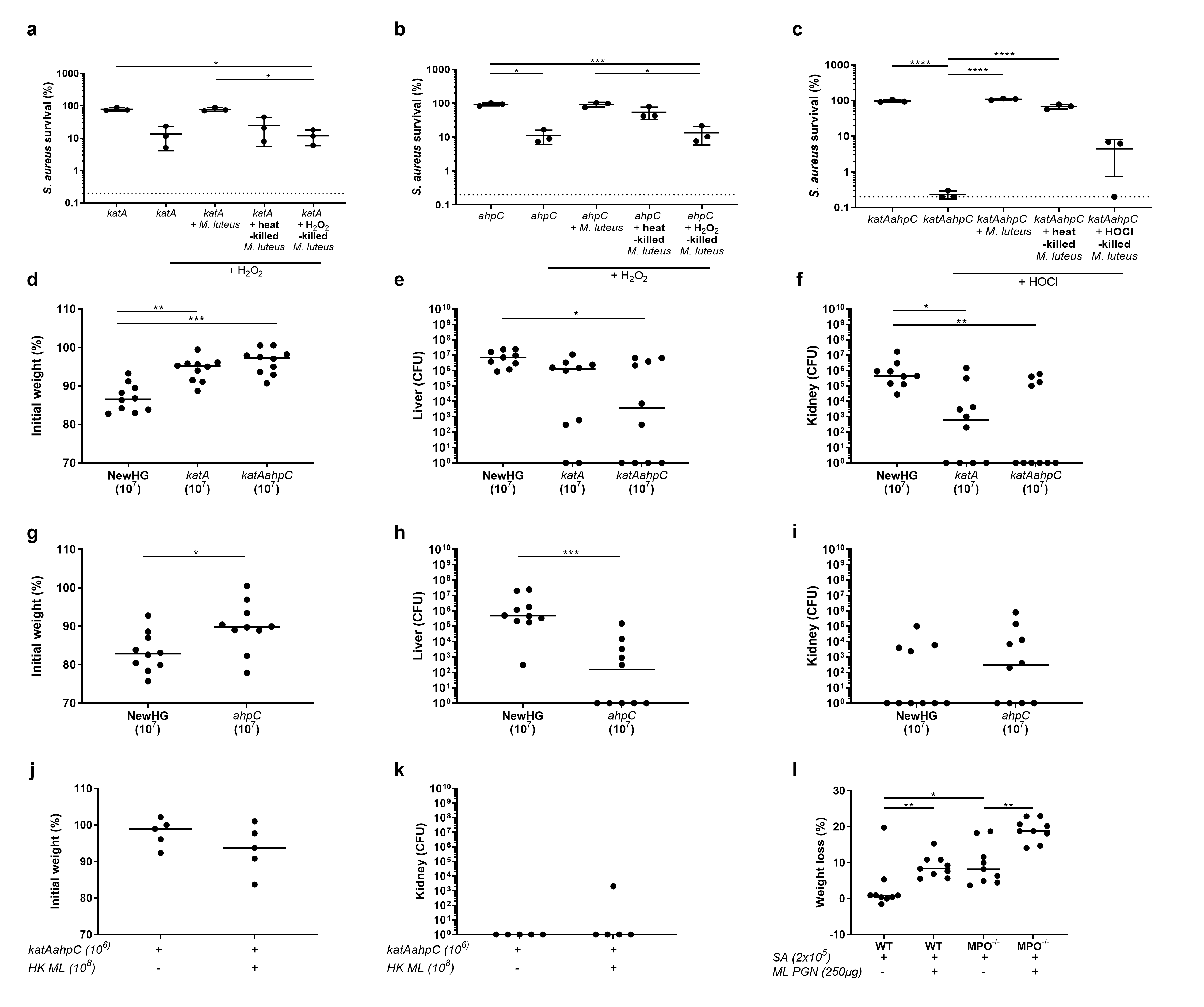

Supplement: S5 Fig — A-B Following hydrogen peroxide exposure, percentage survival of ROS susceptible S. aureus mutants alone (5x10^4^ CFU/mL), or with live M. luteus (5x10^6^ CFU/mL), heat-killed M. luteus (equivalent of 5x10^6^ CFU/mL) or H2O2 killed M. luteus (equivalent of 5x10^6^ CFU/mL), (n = 3, error bars show mean +/- SD, one-way ANOVA tests with Tukey’s post hoc test): A katA, *p<0.05 B ahpC, *p<0.05; ***p<0.0007 C Following sodium hypochlorite exposure, percentage survival of S. aureus katA ahpC alone (5x10^4^ CFU/mL), or with live M. luteus (5x10^6^ CFU/mL), heat-killed M. luteus (equivalent of 5x10^6^ CFU/mL) or ROS killed M. luteus (equivalent of 5x10^6^ CFU/mL), (n = 3, error bars show mean +/- SD, one-way ANOVA test with Tukey’s post hoc test), ****p<0.0001 D-F Injection of high dose (1x10^7^) of S. aureus control, katA, katA ahpC into mice (n = 10 per group, median value shown, Kruskal-Wallis tests with Dunn’s post-test): D weight loss, **p<0.009; ***p<0.0003 E liver CFU, *p<0.05 F kidney CFUs, *p<0.05; **p<0.01 G-I Injection of high dose (1x10^7^) of S. aureus control and ahpC into mice (n = 10 per group, median value shown, two-tailed Mann-Whitney tests): G weight loss, *p<0.05 H liver CFU, ***p<0.0002 I kidney CFU J-K Co-injection of low dose (1x10^6^ CFU) S. aureus katA ahpC with heat-killed M. luteus (equivalent of 1x10^8^ CFU) into mice: liver CFU (n = 5 per group, median value shown, two-tailed Mann-Whitney tests): J weight loss K kidney CFU L Co-injection of low dose S. aureus (SA 2x10^5^ CFU) with M. luteus PGN (ML PGN 500 μg) into wild-type (WT) control mice or MPO-/- mice: weight loss (n = 9 per group, median value shown, individual two-tailed Mann-Whitney tests), *p<0.05; **p<0.004. (TIF) [file ppat.1009880.s005.tif]

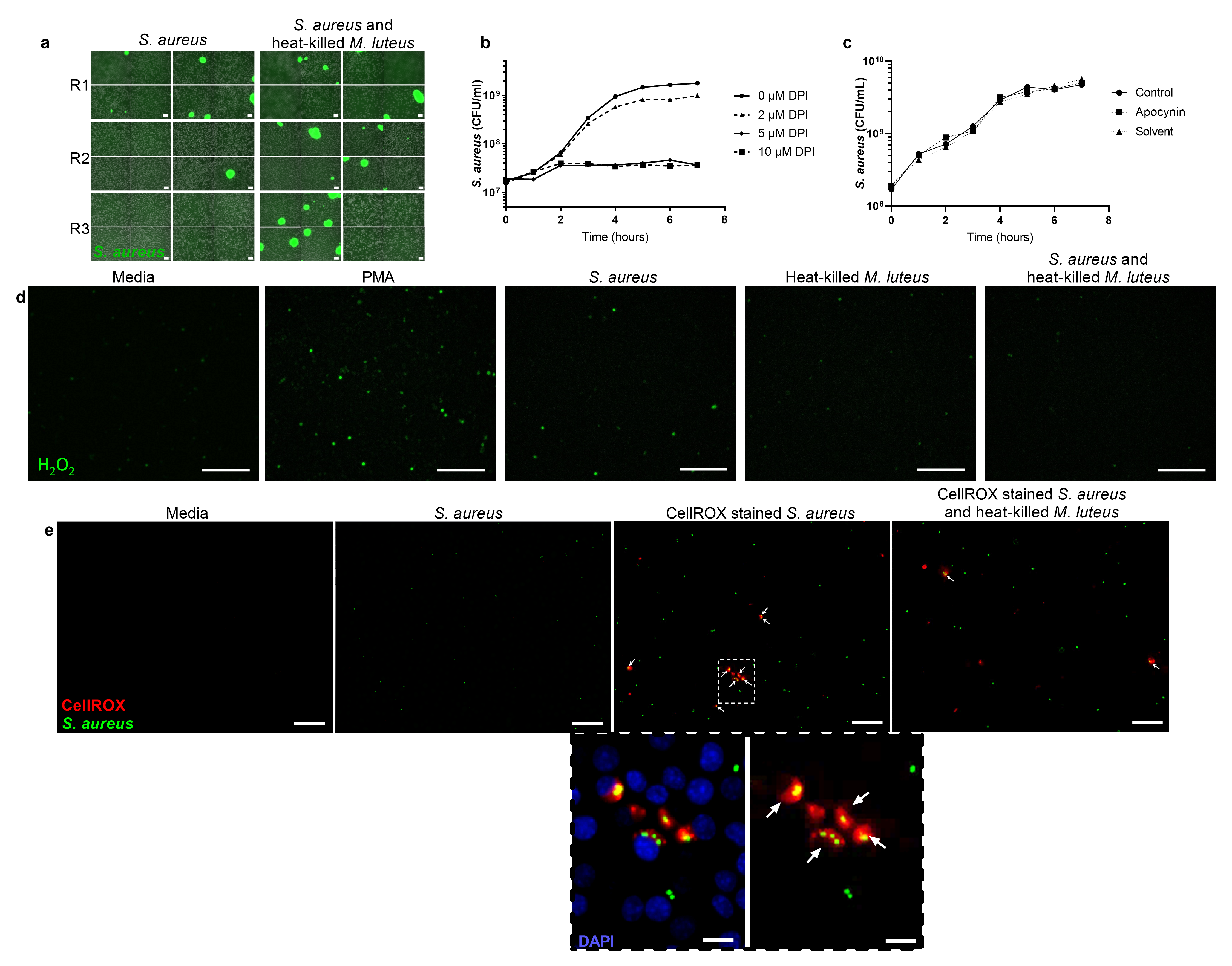

Supplement: S6 Fig — A Representative images of human MDMs infected with GFP S. aureus (MOI 5) in the presence or absence of heat-killed M. luteus (MOI 50), showing number of GFP S. aureus masses observed (n = 3, individual images show a single field of view, 4 per well), scale 100 μm B S. aureus-GFP growth curve in the presence of 0, 2, 5 or 10 μM DPI C S. aureus-GFP growth curve in the presence of 500 μM apocynin or solvent control D Representative images of RAW264.7 cells in the presence of Hydrop (1 μM) (green) incubated alone (media), treated with PMA (200 nM), or infected with S. aureus (MOI 5), heat-killed M. luteus (MOI 50), or both S. aureus and heat-killed M. luteus, scale 20 μm E Representative images of RAW264.7 cells incubated alone (media), infected with S. aureus (MOI 50), CellROX-stained S. aureus (MOI 50) or CellROX-stained S. aureus (MOI 50) and heat-killed M. luteus (MOI 50). RAW264.7 nuclei were stained with DAPI (blue), S. aureus was labelled by GFP expression (green) and, upon oxidation, by CellROX (red). Arrows indicate co-localisation of GFP S. aureus with CellROX signal, implying oxidation of bacteria, scale 50 μm. White box indicates area of increased magnification, scale 10 μm. (TIF) [file ppat.1009880.s006.tif]

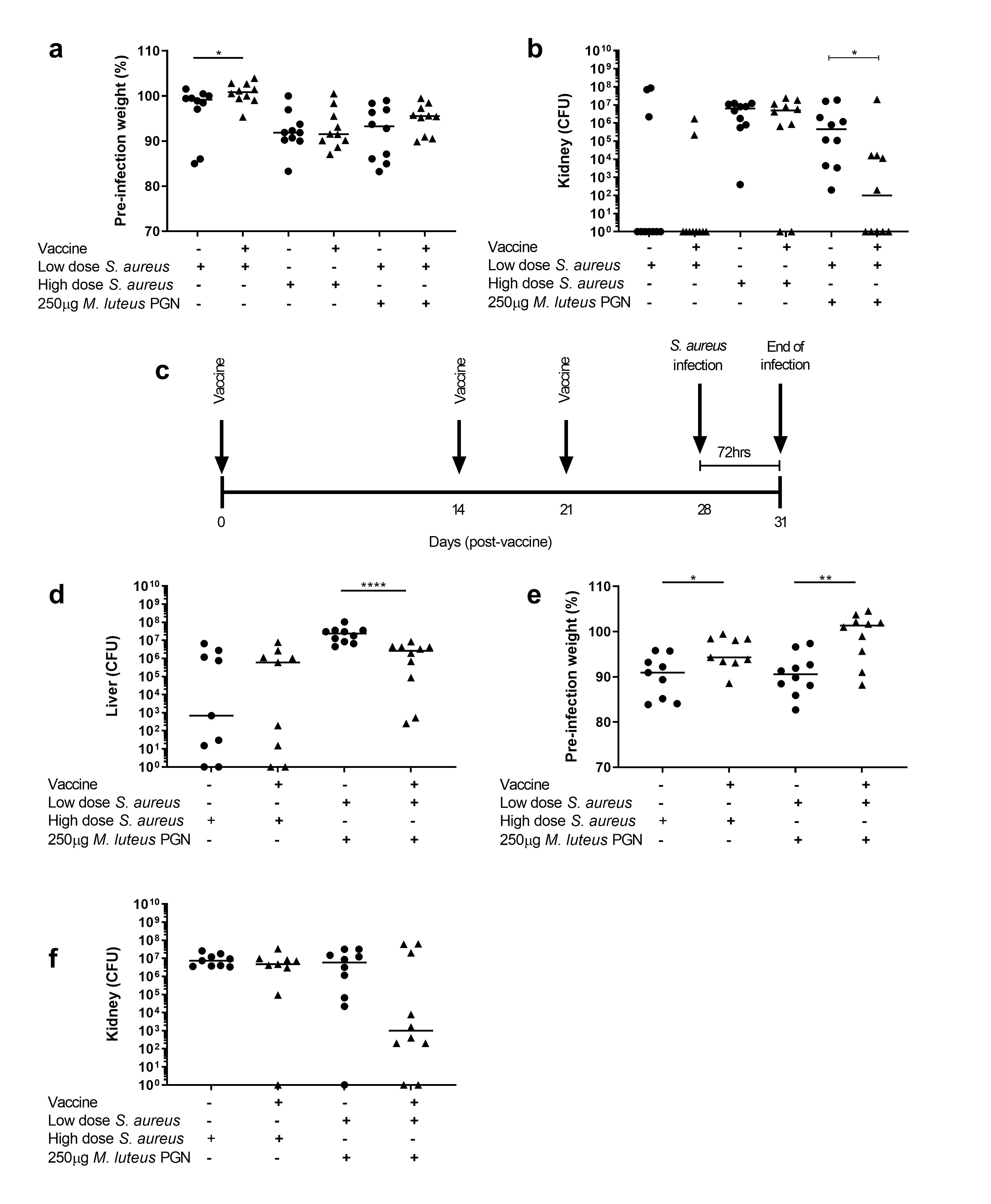

Supplement: S7 Fig — A-B Mice were vaccinated subcutaneously on day 0, 14 and 21 with vaccine (1 μg ClfA, 50 μg CpG and 1% w/v Alhydrogel, triangles) or PBS control (circles). Day 28 post-vaccination mice were intravenously injected with low dose S. aureus (1x10^6^ CFU), high dose S. aureus (1x10^7^ CFU), or both low dose S. aureus and 250 μg M. luteus PGN (n = 10 per group, median value shown, two-tailed Mann-Whitney tests) A weight loss, *p<0.05 B kidney CFU, *p<0.05 C-F Mice were vaccinated subcutaneously on day 0, 14 and 21 with vaccine (1 μg ClfA, 50 μg CpG and 1% w/v Alhydrogel, triangles) or PBS control (circles). Day 28 post-vaccination mice were intravenously injected with high dose S. aureus (5x10^6^ CFU), or low dose S. aureus (5x10^5^ CFU) and 250 μg M. luteus PGN (n = 10 per group, median value shown, two-tailed Mann-Whitney tests) C diagram of experimental protocol D liver CFU, ****p<0.0001 E weight loss, *p<0.05; **p<0.006 F kidney CFU. (TIF) [file ppat.1009880.s007.tif]

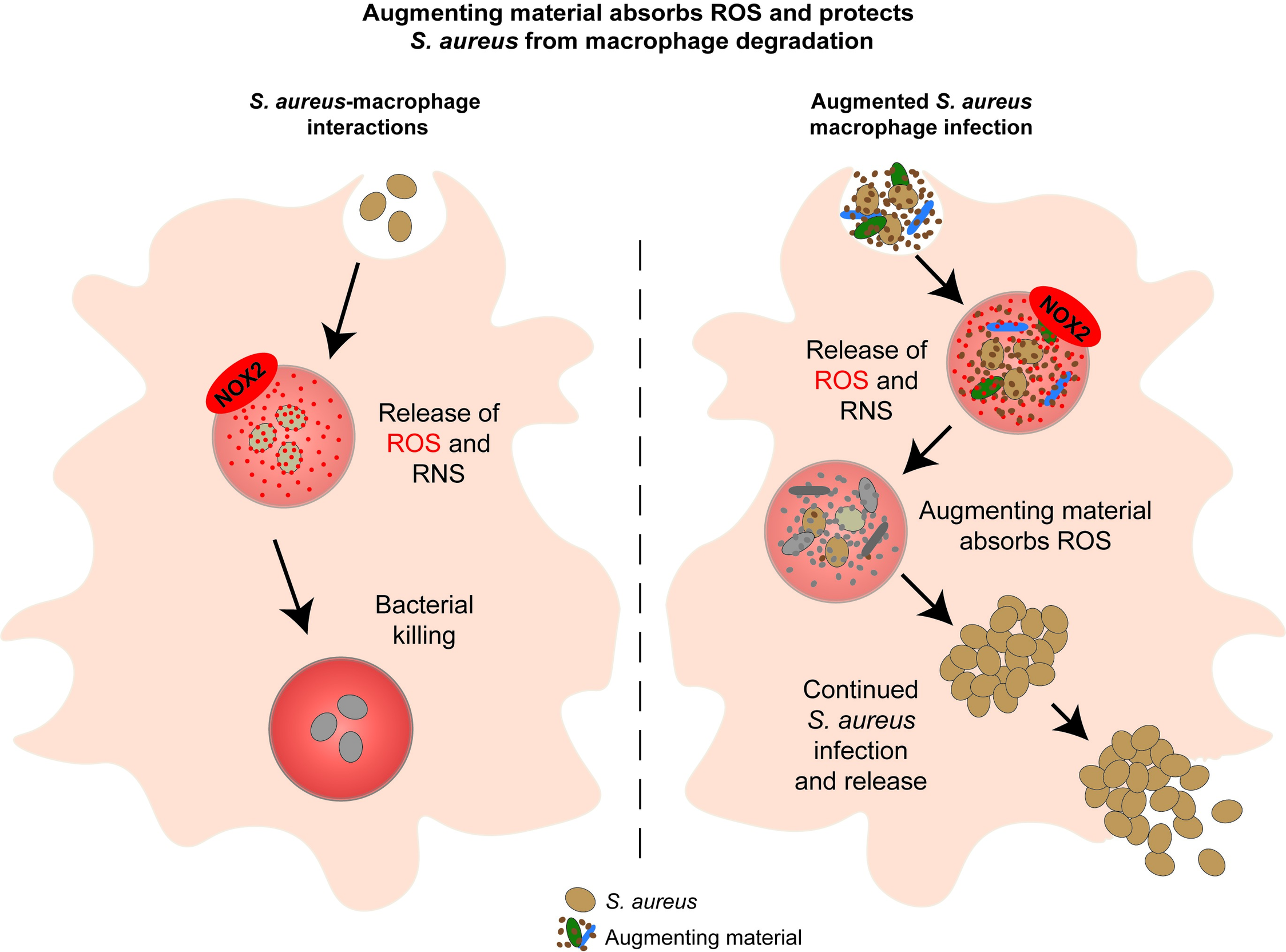

Supplement: S8 Fig — Diagram highlighting how augmenting material protects S. aureus from ROS in the phagosome of macrophages. The left-hand side demonstrates non-augmented S. aureus infection, resulting in ROS mediated bacterial killing. The right-hand side shows an augmented S. aureus infection, highlighting how the presence of augmenting material in the same phagosome as S. aureus results in reduced S. aureus killing due to inactivating ROS produced in the phagosome. Survival S. aureus is then able to proliferate and escape the macrophage. (TIF) [file ppat.1009880.s008.tif]
