# Supplementary material for: Commensal bacteria augment Staphylococcus aureus infection by inactivation of phagocyte-derived reactive oxygen species
Source: PLoS Pathog. 2021 Sep 16;17(9):e1009880. doi: 10.1371/journal.ppat.1009880 (PMC8478205; doi:10.1371/journal.ppat.1009880)
Supplement: Attachment — Submitted filename: Gibosn et al Replies to Comments.docx [file ppat.1009880.s009.docx]

*PPATHOGENS-D-21-01226 Gibson et al.*

*Replies to Reviewers Comments*

*As indicated by the comment of the reviewers, the data in the manuscript are of substantial interest and well presented. Each of the reviewers has a number of concerns and/or clarifications which must be addressed. In particular, reviewer 1 asks, rightly so, whether just increasing the dose of S. aureus itself would similarly function as a sponge for ROS - and why this is not the case. Similarly, rev 3 makes important points regarding the actual mechanism of ROS "neutralization" and where this occurs. Overall, the concept that macrophage generation of ROS is critical in S. aureus clearance is important and this demonstration of heterogeneous augmentation is a nice contribution.*

We thank the editors for their detailed consideration and the opportunity to respond to these comments. We have answered the specific comments from the reviewers below.

*Reviewer Comments (if any, and for reference):*

*Reviewer's Responses to Questions*

***Part I - Summary*** *Please use this section to discuss strengths/weaknesses of study, novelty/significance, general execution and scholarship.*

*Reviewer #1: In this study the authors extend upon their earlier work that documented a process referred to as augmentation whereby skin commensals along with peptidoglycan can enhance the pathogenesis of S. aureus. The authors provide evidence that this process occurs through ameliorating the ROS/killing mechanism of macrophages. This can occur with live and heat killed preparations as well as peptidoglycan. This aspect of the study is well executed. The other elements of the work pertaining to what is required for augmentation and how this inhibits ROS activity is less developed. Other than a multitude of species tested, it is not known if additional S. aureus or HK S. aureus would cause this augmentation phenomenon or the scope of different bacterial components in protection S. aureus from ROS damage. If indeed the augmenting material is just acting as a decoy to ROS, this is a straight forward mechanism, in the sense that more cells will require more time to be killed. Both of these points need some further investigation, along with the vaccination data at the end which doesn’t add to the mechanistic data presented regarding the ROS aspect of the study.*

*Do additional S. aureus or HK S. aureus augment?* We have previously demonstrated that both increased live or dead *S aureus* lead to greater levels of infection (Boldock et al., 2018). The sepsis model is dose dependent as has been found in a number of studies (Pollitt et al., 2018).

*More cells will require more time to be killed?* This is an interesting proposal but we would suggest that the necessity for co-inoculation of *S. aureus* and augmenting material leads to their co-localisation in the macrophage. The ROS burst is finite and so if the *S. aureus* are protected during this burst of killing capacity then they can then potentially go on to proliferate and escape.

*The vaccination does not add to the mechanistic data.* We would agree with this statement. Vaccine development against *S. aureus* has failed over many years, at least in part as the animal models do not reflect human infection. Our augmentation model goes a little way to rectify this as augmentation is likely to be part of the natural infection phenomenon. The demonstration that vaccination can be efficacious using the augmented scenario sets the scene for potential future mechanistic studies.

*Reviewer #2: Could it be that S. cervisiae did not augment S. aureus counts in the liver and S. pneumoniae and P. aeruginosa those of E. faecalis, because these three species may target other organs or body districts. Could authors provide complementary counts of other organs and blood for the supplementary materials?*

As requested, we have added additional organ data from the experiments mentioned where available. To accommodate the extra data, we have split Fig. S1 into two separate supplementary figures: Fig. S1 and Fig. S2. This data includes kidney, lung, heart and spleen CFUs for *E. faecalis* (Fig. S1), kidney CFUs for *S. cerevisiae* (Fig. S1), and kidney CFUs for augmentation of both *S. pneumoniae* and *P. aeruginosa* (Fig. S2). *E. faecalis* was significantly increased in numbers in the lung when co-injected with live *M. luteus*, and in the kidneys, spleen and heart when high dose *E. faecalis* was co-injected with *M. luteus* PGN. However, in all cases *E. faecalis* was not increased to levels exceeding the inoculum. In none of the other cases was any evidence for augmentation observed.

*The hypothesis of augmentation envisages that in each phagosome of each phagocyte the augmenting material co-localises with S. aureus or S. aureus katA aphC. For example in the case of the liver samples (time of sampling in fig 1 not defined) which might be at 72h it is not clear if authors hypothesise that liver macrophages did take up at time 1h post-infection one S. aureus cell and one M. luteus and that both remained together within the same macrophage for 72h or if authors hypothesise that at each cycle of liver macrophage infection, which might occur each 6h, each successive event is determined by a colocalization of S. aureus and M. luteus. There might also be the hypothesis that the colocalization is necessary only for the first moment and that from then on S. aureus is able to start the successive phases of disease (not defined here) also, without needing the help of the mcirocossus. The last paragraph of the discussion nicely addresses this, but authors should provide references for the discussion of their hypotheses.*

This is exactly what we envisage in that it is at the very outset of infection that augmentation occurs. This allows an increased number of escape events from macrophages, which sets up a greater chance for systemic infection to occur, as discussed in (Boldock et al., 2018).

*Please define time of sampling (i.e. liver) of all animal experiments (i.e. Fig 1 and 3).*

All figure legends have now been updated to include the time of sampling for *in vivo* experiments (Fig. 1, 2, 4 and 6).

*Figure 2i authors should give a quantitative or at least semi-quantitative analysis which shows if the phagosome shown in 2i was the only one showing effectively colocalization or if this was the case in many phagocytes.*

A panel has been added to the supplementary data (Fig. S3e) as suggested, showing quantitation of zebrafish macrophages containing *S. aureus* alone, PGN alone, or *S. aureus* co-localised with PGN.

***Part II – Major Issues: Key Experiments Required for Acceptance***

*Reviewer #1: It was shown with different pathogens that they could all augment the S. aureus infection. Beyond what the authors have shown before with PGN and other skin commensals, this does not add further mechanistic data. Can other PAMPS also augment the response? Alternatively, is there a generalized host signaling pathway that is stimulated by all the different species tested that influences the outcome. Does adding back additional S. aureus PGN also augment?*

We have previously shown that receptor-mediated signalling pathways (NOD1, NOD2, Myd88, inflammasome) were not essential for augmentation to occur (Boldock et al., 2018). We have also previously demonstrated that *S. aureus* PGN can augment infection (Boldock *et al.*, 2018). Furthermore, PGN was unaffected in ability to augment regardless of whether lipoproteins or LTA were also present (Boldock et al., 2018).

*Is the augmentation material just acting as a sponge/decoy to the immune system and reactive oxygen species? The data presented in figure 3 seems to support this. If this is the case, wouldn’t just adding heat-killed S. aureus or even more S. aureus cause the same effect? If there is a finite ability of the immune system and/or ROS to deal with bacterial cells adding more would act to quench the activity.*

This is exactly what we suggest is happening. It is the events at the very point of infection that have a crucial role in determining the outcome. That is why we believe augmentation to be important and highlights the gatekeeper role that the phagocytes play in *S. aureus* disease.

*It is known, and acknowledged that murine models of S. aureus while showing promise with vaccines have yet to translate to human studies. While it was interesting to observe that the vaccine used only appeared to cause an effect with the augmented sample, it would be expected based on prior studies that the formulation used would also protect against the standard challenge.*

Over the years we have had a lot of experience with *S. aureus* vaccination experiments and have found them to be very condition dependent (exact vaccine formulation, infection model etc). This is also exacerbated by the somewhat stochastic measure of infection correlates. For instance, kidney numbers are notoriously varied across subjects. The augmented model gives much tighter numbers in terms of CFU as a measure of disease outcome, especially in the liver. Thus we were not overall surprised that the vaccine in the control infection did not protect but the protection in the augmented infection suggests that this maybe a useful model at the outset of vaccine development.

*In figure 4b the WT control is missing. The levels of S. aureus survival of the katAahpC mutant look similar to what was seen with WT in other experiments, without this control, the mutant’s inherent attenuation cannot be confirmed.*

WT data has been added to Fig. 4b for comparison. Although the WT and *katAahpC* *S. aureus* have similar levels of survival *in vitro* in Fig. 4b, Fig. 4c shows considerable attenuation *in vivo*. Cosgrove et al. 2007 showed a significant difference in the H_2_O_2_ resistance of *katAahpC* compared to WT *S. aureus,* however, this was using CT-BHI media rather than PBS, and the difference between strains was negligible at 1 h.

*Figure 3, much of the data is only an n of 3. It is not clear if this data is the accumulation from multiple experiments, or if the experiment needs reproducing.*

The data are from at least 3 biological replicates as is standard for such *in vitro* assays and provides the data for statistical analysis. A sentence has been added to lines 528-529 of the materials and methods to explain this.

*Reviewer #2: Availability of microscopy of early liver samples showing colocalization would strengthen the data.*

We have previously demonstrated the colocalization of *S. aureus* with augmenting material in the liver Kupffer cells of infected mice, using real time, intravital microscopy (Boldock et al*.*, 2018).

**Part III – Minor Issues: Editorial and Data Presentation Modifications**

*Reviewer #1: In figure 2, the color that is used to track PGN is not noted. It is also hard to see if the PGN added is indeed co-localised with S. aureus.*

The legend to Fig. 2 now highlights the colour used for PG visualisation (blue). We agree that it is difficult to see in the reproduced images which is why we have the grey scale insets. These are now explained explicitly in the legend (l. 858-859).

*Several pertinent comparisons in the data were lack statistical testing, such as Figure 3b is S. aureus alone different to S. aureus with PGN in the present of H2O2? Likewise, 3f, comparing the different groups with H2O2 treatment.*

The requested statistical analysis has been added to Fig. 3b and 3f.

*In figure 6, the level of infection with both the low and high doses of S. aureus without vaccination were less than what was observed in earlier experiments.*

This pertains to the variability in infection outcome in the non-augmented model, which is why we include the control in each (See Fig. 1a, b, c). See also the reply to the vaccination comment above.

*The authors may wish to include in their discussion why they see protection/augmentation with heat-killed preparations under some conditions but not others.*

As suggested we have added a section to the discussion, to compare the level of protection observed under different conditions and the comparability between heat-killed cells and purified PGN (l. 358-366).

*Reviewer #3: 1. While it is accurate to state the augmentation phenotype is not observed in transgenic mice lacking a functional NOX2 (as shown in Fig. 4, Boldock et al., Nat Microbiol, 2018), the text related to the requirement for NOX2 in augmentation needs clarification in the current manuscript. Simply put, the shielding phenotype conferred by commensal microbes would not be needed to promote optimal growth in mice lacking NOX2. The growth of SA alone is augmented already because phagocytes in Cybb-/- mice lack the ability to produce ROS, which contribute to bacterial killing. This is the likely reason for a lack of difference between SA alone and SA + PGN in Cybb-/- mice (Fig 4b) in the Boldock et al. Nat Microbiol paper. This point is underscored further by the much greater RFL per mm2 in Cybb-/- mice compared to that in the wild-type mice. These findings support the authors conclusions in the current manuscript, but not for the reasons implied by the text (lines 156 and 187). That is, ROS do not contribute to the shielding mechanism in vitro, but they are required to illustrate a difference between shielded and unshielded phenotypes. There could also be a difference in mechanism between in vitro assays and in vivo models, although this is beyond the scope of the current studies. Please revise the text to clarify the conclusion related to the role of NOX2, which was not optimally stated.*

We have taken these comments on board and altered the text to be more equivocal about the role of NOX2 as suggested:

l. 157: Macrophages and ROS are implicated in augmentation (12).

l. 188-191: **Since augmentation does not occur in the absence of NOX2**, and Kupffer cells have reduced ROS levels in augmented *S. aureus* infection (12), we hypothesised that inactivation of ROS by augmenting material **could be** the mechanism by which *S. aureus* survival is enhanced with an ensuing increase in pathogenesis.

We have also added a sentence to qualify the association between *in vitro* and *in vivo* data (see reply to comment below).

*2. The assays that test the effects of reactive oxygen species in vitro can be influenced considerably by the assay buffer or media. For example, hydrogen peroxide can kill S. aureus readily in PBS, as shown in Fig. 3. However, the results will be different in an assay buffer that permits sustained S. aureus metabolism and/or replication, even if minimal (e.g., DMEM with glucose). Also, hydrogen peroxide (at millimolar concentrations) can have little or no impact on S. aureus survival in a rich media such as TSB. This is likely due to the interaction of ROS with components of media (a mechanism perhaps similar to the “shielding” reported here), and the ability of S. aureus to rapidly produce molecules needed for defense against ROS. This does not occur in PBS. I understand that these scenarios are different. The PBS assay addresses whether ROS have the potential to kill S. aureus, whereas assays performed in tissue culture or bacterial growth media address whether ROS kill S. aureus under conditions that permit sustained S. aureus metabolism and growth. Some additional text might be help clarify this potential issue with in vitro assays.*

We agree and have added the sentence below to the Discussion to highlight this point (l. 369-372).

The *in vitro* data was obtained in an environment very different from that experienced by the bacteria inside phagocytes, let alone *in vivo*, therefore it was important to make analyses in these more complex milieu.
